# Playing “mental judo”: Mapping staff compassion in Canadian federal prisons

**DOI:** 10.1177/14624745241237173

**Published:** 2024-05-03

**Authors:** Katarina Bogosavljević, Jennifer M Kilty

**Affiliations:** 6363 University of Ottawa, Canada

**Keywords:** compassion, emotional labor, prison staff, Canada, prisons

## Abstract

Prisons are inherently emotional environments where both staff and prisoners engage in a continuous process of emotion management while working and living in carceral spaces. This paper explores how Correctional Service of Canada (CSC) values and norms shape how predominantly nonuniformed staff manage compassion inside the prison environment. This includes when and to whom they are allowed to express compassion, when they need to hide or suppress the expression of compassion, and how expressing compassion toward prisoners can elicit feelings of disgust among some staff. We argue that, in the emotional arena that is prison, compassion is (re)configured into an individualized and compulsory emotion by way of CSC's organizational emotion culture that emphasizes punishment and control (the security-care nexus) rather than a transformative act that helps to resist the harms of incarceration and encourages healing. Compassion thus becomes a disciplinary apparatus whereby staff self-discipline as they alter their own emotional orientation toward their work, prisoners, and other staff and as a practice to collectively surveil, evaluate, and regulate one another. We contend that compassion bound to questions and practices of security stifles rehabilitation in this environment and that health and other care work must be reintegrated into community settings.

## Introduction

You’re tapping into emotions… You’re tapping into their beliefs and their ignorance and their biases and their misogyny and their anti-authoritative beliefs. It's just a lot. … *I call it mental judo* … being able to coordinate that whole operation. I think it takes a lot of skill and a lot of balance, and at the same time you have to create the rapport.

(Amy, programs officer)

This paper explores how Correctional Service of Canada (CSC) culture shapes how staff manage compassion inside the prison environment. This includes when they hide or suppress expressions of compassion, to whom they are allowed to show compassion, and how acting compassionate toward prisoners can elicit feelings of disgust among some staff. Understanding how staff engage in emotion management, something that is scarcely explored in the literature (Crawley, 2004a, 2004b; Tracy, 2005, 2008), requires examining emotions as productive forces that “endow ‘others’ with meaning and value” (Ahmed, 2004: 4). Emotions are important to consider as they are an integral aspect of carceral life (Bosworth, 2019; Brown, 2012; Crawley, 2004a, 2004b; Nylander et al., 2011; Nylander and Bruhn, 2021). Indeed, we know that prisoners experience emotional hardships while incarcerated (Crewe et al., 2014) and feelings of frustration, anger, resentment, sadness, fear, anxiety, and hopelessness are commonplace. While happiness, hope, and joy are less common, prisoners do report having these feelings particularly when showing solidarity to one another (Fayter, 2022).

Building on Hochschild's (1983) pioneering work on emotional labor, we shift away from understanding emotions as something that occurs “inside” a person and that work toward an organizationally related outcome to explore how “power circulates in and around organizations” by way of discourses that structure, “produce and reproduce particular meanings” (Fineman, 2010: 25) that shape people's feelings about their work. Nowhere is this more evident than in prison where wellness orientated staff experience emotive dissonance (Walsh, 2009), meaning a difference in one's “true” versus displayed feelings (Hochschild, 1983), as they attempt to care for prisoners while working in an environment dominated by an ethos of security, punishment, and suspicion and that requires staff to act detached.

While the limited existing work on emotional labor in prisons tends to focus on correctional officers (COs), this paper uniquely includes the experiences of nonuniformed staff. We are interested in how compassion is a compulsory emotion within CSC's organizational emotion culture. Within this security-care nexus, compassion operates as a disciplinary apparatus both in the sense of self-discipline, where staff alter their own emotional orientation toward their work, prisoners, and other staff, and as a practice to collectively surveil, evaluate, and regulate one another if it is expressed inappropriately. We build on work that details how security and care logics conflict in prison such as when this nexus makes meeting the demands of palliatative care difficult for aging prisoners (Marti et al., 2017); subsumes harm reduction measures like needle exchange programs under surveillance networks that fail to support people who use drugs (Michaud and Van Der Meulen, 2023); and co-opts restorative justice conceptualizations of relationships and values by merging them with regime expectations of detachment and separation, “that is, no-care” (Elliott 2007: 205). We heed Brown's (2012: 390) argument that, “care exists at a vortex of suspicion” in prisons to show how compassion is bound to questions and practices of security and is closely monitored by institutional personnel.

In what follows, we conceptualize the prison as an emotional arena where compassion might be encouraged but is also villainized such that staff who show compassion are considered suspect of creating possible security threats. After describing the project's methodology, we present two key findings. First, that CSC's culture, norms, and values (the security-care nexus) propagate a compulsory form of compassion which widens the social gap between those who express compassion (staff) and those who receive it (prisoners) because compassion can be used to put others down, elevate oneself, or both (Clark, 1997). We map the intricacies of how compassion is performed by uniformed and nonuniformed prison staff, noting when it is allowed or discouraged and by whom. Second, we challenge the notion that compassion is an inherently “good” emotion, instead showing how compassion operates to bind the disgust and fear that is commonly attached to prisoners to those staff members who express compassion toward them. This is a form of “staining” that occurs in “dirty work,” defined as those tasks, occupations, and roles that are more likely to be culturally perceived as disgusting or degrading, for example, care work, migrant labor, night work, and domestic or other service work (Ashforth & Kreiner, 1999). Given that correctional labor involves many of these job dimensions, it may be understood as a form of dirty work. As staff who show compassion are treated with suspicion by their colleagues, tense collegial working relationships can sometimes push compassionate staff to succumb to the prison's organizational culture, which discourages compassion and can lead to mental fatigue and distress.

### Prisons as emotional arenas

Emotions are the social expressions of our affective experiences; they are the feelings that we perform for others to see (Hochschild, 1979). Emotions are experiences that are “shaped by social structures and the norms and values of the organization” (Fineman, 2008: 1). Emotions are not ideologically neutral or *just* clinical symptoms of trauma or signals located in particular parts of the brain or body; they reflect cultural and political experiences (Ahmed, 2004; Hochschild, 1979) and workplace culture (Fineman, 2008). An emotion culture is a set of feeling rules that is created in any organizational setting. These rules are the “social guidelines” that dictate how we should feel, the depth or degree of said feelings in different circumstances, and the direction and duration of feelings (Hochschild, 1979). Feeling rules are taken from the wider societal emotion culture in which the organization resides and are often adapted by organizations to “create their own codes of emotion propriety” (Fineman, 2000: 3). Staff engage in emotional labor through both surface (outward displays of emotion) and deep acting (the inner work of managing one's emotions and feelings) to abide by the feeling rules of the organization (Hochschild, 1983). Feeling rules in prison govern the everyday interactions correctional staff members have with each other and with prisoners. Prison officers actively marshal well-rehearsed emotion-work strategies to suppress “improper” emotions (Crawley, 2004a) where failure to do so runs the risk of being identified as “someone who is either not ‘one of us’ or not ‘up to the job,’” which signals how emotion management is central to prison governance (Crawley, 2004b: 424). Similarly, COs engage in complex surface acting to be respectful while experiencing perpetual suspicion and being “on the lookout” for prisoners who disobey rules, balancing the correctional ethos of rehabilitation while also being disciplinarians (Tracy, 2005). Feeling rules dictate that COs act aloof to avoid being, or perceived as being, compromised; emotions are thus positioned as a threat to objectivity and the security of the institution.

Prisons are unique emotional arenas (Crawley, 2004a, 2004b; Tracy, 2008) that contain zones where “the normal rules of the prisoner society were partially or temporarily suspended, permitting a broader emotional register than was possible in its main residential and most public areas” (Crewe et al. 2014: 67). These zones are where organizational feeling rules are tested as “emotions are ‘performed’ in a particular context for a particular audience (bosses, collegues, customers, clients, patients, competitors)—people to be influenced or impressed, placated or befriended, repelled or shamed” (Fineman, 2008: 4). Emotive performances are not impulsive, but acted-out through talk and bodily movements that are “aligned to the micro-structure of the situation” (Fineman, 2008: 4). Emotions are important strategic resources that can sustain, augment, or destabilize micro-social arrangments (Fineman, 2008). Nylander et al. (2011) contend that COs tend to have closer relationships with lower security prisoners and those residing in treatment areas who are identified as less dangerous and less likely to threaten institutional security. In these spaces, officers are more likely to engage in deep acting, rather than the surface acting they perfom in higher security spaces. Emotion zones can help prison officers to cope with the impact of the emotional labor they perform in different prison, unit, and wing subcultures (Nylander and Bruhn 2021).

Failure to perform emotions “correctly” may lead to the outing of “emotional deviants” (Thoits, 2012) which can result in cliques forming among security staff and the “caring” professions, such as behavioral counselors and health care workers—creating what we describe as the security-care nexus. Emotional labor can lead to burnout, depression, role alienation, emotional numbness, exhaustion, and identity confusion when there is a disjuncture between displayed emotions and private feelings (Fineman, 2000; Nylander and Bruhn, 2021; Tracy, 2008). Indeed, research on CO mental health in Canada shows that working in the correctional environment can lead to post-traumatic stress disorder, social anxiety, panic disorder, and depression (Easterbrook et al., 2022; Fusco et al., 2021). What remains unexplored in this literature is how staff emotions are “constituted through overlapping discourses of power” (Tracy, 2008: 31) and how the organizational ethos works upon staff emotions. Situating emotions as relational allows us to consider burnout and work-related stress as resulting from structural power dynamics that encourage or discourage expressions of particular emotions, such as compassion.

We focus our attention on how CSC staff perform compassion and how compassion operates politically, culturally, and socially in the “emotional arena” (Fineman, 2008) of Canadian federal prisons. Compassion is a motivational force in that it is “basic acts of kindness and care that create and sustain human social life” (Wilkinson, 2018: 56). Compassion can also be a selfish and privileged act (Wilkinson, 2018). As Berlant (2004: 4) contends, “compassion is an *emotion in operation*… [it] denot[es] privilege: the sufferer is *over there*. You, the compassionate one, have a resource that would alleviate someone else's suffering.” Compassion, like it's sister emotion sympathy, can operate in ways that “underline, intensify, or reverse the degree of inequality” meaning we must be attuned to the “micropolitical aspects of emotional interchanges” which “place” us within microhierarchical arrangements that reflect the everyday minutia of our social interactions (Clark, 1997: 229). Emotional cues or “place claims,” such as anxiety or feeling hurt, identifies our shifting place in these arrangements “often before other kinds of cues come to awareness” (Clark, 1997: 233). As such, it is important to explore how compassion is an “emotional place claim” that reinforces the “microlevel place arrangements and, consequently, macrolevel power arrangments” (Clark, 1997: 235).

Compassion can be a force for social, political, and cultural change, but it has also been mobilized in disciplinary ways. As Arendt (1963/2006: 84) writes, “compassion became the driving force” of many revolutionaries who used violence to achieve sociopolitical change. We suggest that a kind of compulsory compassion (Acorn, 2004) operates within CSC. While staff might perform acts of compassion in their day-to-day activities (e.g., hugging, engaging in personal conversations, using discretionary power to assist a prisoner or to refrain from giving an institutional charge for a minor offence like backtalk or sharing food), their efforts are structured by policies that control and punish. The emotional arena of prison prevents and punishes “restorative compassion” which is rooted in “a vivid awareness of the concreteness and complexity of the inner lives of others” (Acorn, 2004: 138). This entails mutuality, whereby compassion is exercised by all parties, and humility, where we acknowledge our own and other's “implicatedness in a dual condition of human suffering and capacity for renewal” (Acorn, 2004: 140). Such compassion requires respectful dialogue and interaction that involves truth-telling, an exercise that is difficult in carceral environments where prisoners often do not trust and therefore withhold information from staff (Brown, 2012). Indeed, CSC's mission statement emphasizes “assisting offenders to become law-abiding citizens, while exercising reasonable, safe, secure and humane control.” Compassion in this context thus reflects a “strict and stern paternalism to the demand for discipline and responsibility” (Woodward, 2004: 73). If rehabilitation is a sincere correctional goal, it must “give attention to the generative power of a truly imaginative empathy with multiple, culturally contingent meanings” (Brown, 2012: 386).

## Method

Our project examines the affective politics of carceral spaces, with the goal of documenting the emotional geography of federal penitentiary spaces in Canada. The project aims to foreground how people with material experiences of living or working inside federal prison navigate the day-to-day emotional dynamics and politics of prison life. To do this, we conducted *N* = 75 semistructured interviews (57 with formerly federally incarcerated people and 18 with correctional staff). After securing approval from our university's research ethics board, we recruited participants through two approaches. First, team members uploaded recruitment posters to their various social media accounts (notably, on Facebook and Instagram), which was particularly helpful in recruiting correctional staff participants; snowball sampling also helped with recruiting this population. Second, following approval for recruitment from the Executive Directors, we recruited formerly incarcerated participants through nonprofit community-based organizations that work with criminalized people. Staff distributed recruitment materials to service users, who contacted one of three doctoral-level research assistants by phone or email to indicate their willingness to participate and to schedule a time for the interview. Virtual^1^ interviews were conducted between December 2020 and August 2021, on average lasting approximately 90 min.

Following verbatim transcription, interview transcripts were coded using NVivo qualitative analysis software and a thematic narrative coding process (Riessman, 2008) that concentrates on “what” is said rather than how a speech act is organized. Narrative analyses are typically case centered—here we concentrate on the participant group of federal correctional staff members—which includes uniformed (*N* = 2) (i.e., COs or “guards”) and nonuniformed staff (*N* = 16) (i.e., parole officers, program officers, behavioral counselors, nurses, an Indigenous liaison and program officer, volunteers,^2^ a contract employee, a deputy warden, and a prison unit manager).^3^ We begin by examining how the security-care nexus shapes staff efforts to show compassion, followed by a discussion of how nonuniformed staff engage in taint management to maintain collegial working relationships with uniformed staff.

### The security-care nexus and compulsory compassion

While Canada's federal correctional system is touted as a world leader in correctional policy with its focus on rehabilitation (Kilty and Bromwich, 2020), CSC's values and norms limit care by requiring staff to act detached and to not *feel* for prisoners who are chronically regarded with skepticism and distrust. The compassion that operates in Canadian federal prisons is compulsory in that compassion tied to justice is always-already a “discipline and an achievement rather than a spontaneous impulse” (Acorn, 2004: 137). Correctional authorities cannot demand compassion from staff, although they can “encourage, nurture, cultivate and even discipline in a more compassionate sensibility” (Acorn, 2004: 137). Such “politics of personal feeling” (Woodward, 2004: 71) cannot address the injustices faced by prisoners precisely because compassion in prison cannot be “translated into protest at injustice or transmuted into policy to alleviate suffering” (Woodward, 2004: 63). The compassion performed in prison is individualized, such that it is organizationally encouraged in ways that promote assimilation with CSC's organizational emotion culture and ethos of security.

In this section, we explore the tensions between staff members’ understandings of acceptable expressions of compassion in the correctional environment, which highlight how carceral spaces are designed to accomplish seemingly contradictory objectives, namely, punishment and rehabilitation. We conceptualize this is as the security-care nexus, where staff working on the two sides of this yoke often find themselves at odds in terms of how they understand their roles and the work they perform. Participants reported that expressing emotion is difficult and can lead to complications between different groups of employees. As Brittany (nurse) stated: “CSC is a very hard place… just having emotions within this organization is really frowned upon in many ways, and they’re definitely not handled very well.” In other words, the security-care nexus requires a failure to be moved by another's suffering, instead emphasizing a compulsory compassion that operates as an emotional reflex, the feeling of which may be fleeting, rather than an ethical achievement (Acorn, 2004).

We do not mean to suggest that staff do not care, do not desire to care, or that they do not feel a “true” compassion toward prisoners; some staff care quite deeply and face mental health struggles from working in this emotional arena. Tait (2008: 6) contends that care involves “values, practices and attitude” that respect, show interest, and respond to prisoners’ needs by being civil, sociable, bracketing, and offering practical help. Care creates rather than threatens institutional security by diminishing “feelings of powerlessness, isolation, and worthlessness engendered by the prison environment” (Tait, 2008: 6). Briefly, we found that nonuniformed staff display varying care styles and attitudes toward prisoners. Behavioral counselors, nurses, volunteers, contract staff, and one of the COs (Katy) were “true carers” (Tait, 2008, 2011) who developed relationships with, showed empathy toward, and enjoyed talking to prisoners while parole officers, deputy warden, and unit manager reflected Tait's limited and old school carers because they strictly enforced institutional rules and acted paternally toward prisoners. Program officers and the Indigenous liason officer exhibited characteristics of both true and conflicted carers as their professional roles enable a caring attitude. As with all typologies, categorizing participants into these groups is not a prescriptive practice.

Writing about British Immigration Removal Centres, Bosworth (2019: 547) found that officers had a difficult time building meaningful relationships with detainees, where “this rupture, with the other and with their self, can be profoundly painful and destabilizing.” However, when staff perform acts of compassion like hugging a prisoner, it is not inherently because they are standing up to an institutionally punitive culture. As Katy, a former CO who trained at one of Canada's healing lodges^4^ describes:

Your own staff members can either be your biggest allies or your biggest enemies. Everybody knew I was a hugger. I embraced that and I was fine with that. My treating the offenders [well] is not necessarily because I’m coddling them. It's because it's a reflection of who I am… everybody needs to be treated with dignity and respect as a reflection of who I am. Being civil and polite, being appropriate and not treating them like pieces of shit is because of who I am.

Katy's personal morals clearly shape her work morals, which in practice operate in ways that resist institutional norms. She justified her actions, stating: “Some of the staff members would misunderstand why I’m a hugger… I want the best for [prisoners], because …it's the best for us.” This explanation reflects how the prison's organizational culture infuses the thoughts of even those staff members whose personal values counter its norms. It also shows how the security-care nexus individualizes compassion such that it operates as a representation of power relations in which marginalized people are dominated by “institutionally over-privileged individuals” (Wilkinson, 2018: 57). We are reminded here of Brown's (2012: 393) argument that “empathetic engagements … are largely disciplinary, where self-sufficiency and emotional control are both points of social control and resistance, always privileging individualized efforts over social or political action.”

Interestingly, staff who are somewhat distanced from the institutional culture because they work as contract employees or volunteers described standing up to management despite their full-time colleagues being suspicious of them for caring “too much.” Henry ran a monthly support group in a maximum-security prison and communicated that there is a balance he must strike in his advocacy work because security staff question his role.

We have to balance the rules of the institution and also being like, “hey, no, this is a violation of their human rights and we’re going to advocate on their behalf.” We’ve had meetings with the warden or with staff, troubling this idea that these people are so inherently violent that… they almost have a spoiled identity… we know that everybody in the group has been convicted of murder or some sort of terrible violent assault or conspiracy to commit. But they’re almost like, “why is there people in the community that want to come in and help these people”?

Similarly, Dale, a college educator on contract with CSC, described challenging COs who intrude on his class,

When I’m in my classroom, that's my domain and I’m very territorial about that. When correctional officers just walk in, I have a problem with that. I will let them know that. In front of the inmates. And of course, that does not go well… I’m not trying to minimise, but I certainly express in an assertive way that I don’t appreciate the disruption… [they say] “Oh, you are the guy … we know who you are.” No, you don’t. It's a small community among the workers and some people react in a very negative way [to] the way I conduct myself in my classroom.

For contract staff, their distance from CSC seems to afford them the ability to confront some of the injustices prisoners experience and resist the bullying tactics employed by COs. For Dale, showing care and compassion toward his students meant directing a stern exterior toward uniformed staff to protect the integrity of the space in which he interacts with prisoners, which often resulted in being shunned by uniformed staff. This suggests that Dale's actions are understood by some security staff, certainly those that match Tait's (2008, 2011) “old school” and “conflicted” officer caring styles, as crossing a professional boundary. Being an advocate requires a delicate balancing act because, as Henry explains, “we could only talk positive about CSC” or risk losing the ability to host the support group inside. Threats of removing privileged access for contract and volunteer staff works to control the level of compassion these staff members may be willing to show.

The different job requirements of uniformed versus nonuniformed staff are most obvious when considering the efforts of those who provide health care. The nurses and counselors we spoke with identified the dissonance between the governing logics of care and security in this environment as one of the biggest challenges to doing their jobs. This difficulty arises with respect to ascertaining prisoner motives and the ways security staff engage with them; health care staff have regular contact with COs who are responsible for bringing individuals to and from the medical wing for assessment and treatment.

When they enter the health care building, they are patients first; security is something we do need to keep [at the] forefront, [but] it's very important to put humanity first. These are human beings that for a variety of reasons have ended up here. We say patient. We don’t say convict because that's not the lens that's necessary for us… The way we speak to the guys is very different than the officers. We need to have a different level of trust to provide quality health care. We need them to feel safe. To tell us what's really going on, to ask embarrassing questions. It's not the same as providing health care in a community setting because all the stuff they teach you in nursing school, you know, divulge a bit about yourself to make people comfortable, that's off the table. (Margaret, nurse)

Margaret highlights how the emotional display rules and behavioral expectations of the carceral environment are shaped by the governing security and risk logics that dominate these spaces, which subsequently alters the approach that health care staff take when providing care. While health care staff may naturally feel compassion toward their patients, their ability to express it is compromised by the security focused nature of this space. A quote from Alyssa (nurse) is as stark as it is revealing about one's abilty to express compassion in this emotional arena: “I had to be very regimented in what I was saying and almost cold. I had to be very matter of fact. I did not always feel the freedom to make a human connection or to have that therapeutic relationship.” The compassion that nonuniformed staff perform is more aligned with the feeling of pity which “denotes the feeling of empathetic identification with the sufferer”—akin to Tait's (2008, 2011) “limited carers”—while a “true” compassion “refers to the feeling accompanied by action” (Zembylas, 2013: 507), something that Tait's (2008, 2011) “true carers” come closest to resembling in the suble ways they resisted negative sterotyping of prisoners. The security-care nexus limits care precisely by requiring a failure to be moved or nonfeeling. For instance, Jason (Indigenous liason officer) acknowledged that when he enters the segregation unit, “I’m a little bit different…because [prisoners are] there for a reason…they chose to place themselves [there], which is not good behaviour.” Starting from the belief that punitive isolation is deserved makes it easier for staff to act detached, which demonstrates how the prison's organizational norms and values encourage a failure to be moved, to not feel for prisoners, and in particular those who do not abide by institutional rules.

Compassion rests on the presumption that suffering is universal, that we can feel the pain of others, and by doing so experience a “feeling of self-satisfaction” because we are “doing the right thing” (Woodward, 2004: 71). While this feeling is supposed to move us to action to address injustice, instead it “returns us to a private world far removed from the public sphere” (Woodward, 2004: 71). As Whitebrook (2002: 530) argues, “suffering …is liable *prima facie* to invoke a response; however, reflective judgement may quickly follow and then initial identification with suffering or vulnerability may be modified, even if not entirely negated.” Applying this critique of compassion to correctional work, we can understand how the security-care nexus engenders fleeting feelings of pity for the sufferer that move toward passivity or “doing the bare minimum.” As Arendt (1963/2006) argues, witnessing the “spectacle” of another's suffering can be right in front of us yet we fail to see or be moved by it. This recounts Foucault's (1979: 256) powerful argument that prison is a disciplinary apparatus where the “power to punish, which no longer dares to manifest itself openly, silently organizes a field of objectivity in which punishment will be able to function openly as treatment and the sentence be inscribed among the discourses of knowledge.” In this environment, one's ability to respond to suffering is stifled by the neoliberal risk logic that is the central feature of Canadian prison culture (Hannah-Moffat, 2005). Compassion is subsumed under security, surveillance, and punishment ideals that do not promote prisoner well-being or relationship building, but demand staff follow institutional rules without discretion, which encourages noncaring acts like giving institutional charges for things like backtalk, sharing or trading food, or not allowing prisoners to make a phone call (Fayter, 2022).

### Compassion is dirty work: Taint management among nonuniformed staff

Thus far we have demonstrated how organizational rules shape the ways staff perform and manage the expression of compassion toward incarcerated people. One of the biggest hurdles nonuniformed staff face in this regard is borne from the security-care nexus that overestimates risk without adequate consideration of prisoner needs (Hannah-Moffat, 2005; Maurutto and Hannah-Moffat, 2006). Given these two different mandates, it is perhaps unsurprising that staff feel that “there's a division. There's a certain culture that the guards have, and then we’re the program people. When I first got into…work, [colleagues] said, “they’re going to call you a con hugger” (Amy, program officer). We concentrate on the underexamined dynamic between different groups of correctional staff, by showing how expressing compassion toward prisoners binds the negative emotions commonly attached to them (disgust and fear) to (predominantly nonuniformed) staff. In this way, compassion is constituted as dirty work by uniformed staff, which results in nonuniformed staff feeling “othered” by this kind of “staining.”

Grounded in Douglas’ (1966/2002) pioneering thought on how perceptions of cleanliness entail establishing boundaries that separate the pure from the polluted, we contend that correctional norms that position criminalized people as innately manipulative and untrustworthy are symbolic codes for disgust (Simpson et al., 2012). The proximity between criminalized people and staff who must develop rapport with them to perform support, health, and other care work effectively taints these staff members and care work. In the carceral context, “dirt” is bound up with amorality, social (dis)order, and the prevailing correctional norms and values that are structured by the security and risk logics that govern these spaces. Certain prisoner groups, particularly those convicted of sexual offences, occupy lower positions in the prison social hierarchy as a result of the moral stains of their crimes and are routinely thought to present greater security risks, particularly to female staff (Ievins, 2019, 2023), despite displaying more compliant behavior while incarcerated (Blagden et al., 2019). The visible but unseen work of program officers, support workers, and those working in the health professions has a low cultural priority among security staff, often highlighted only when a staff member crosses a professional boundary by breaching policy or codes of conduct (Kilty, 2018; Simpson et al., 2012; Sponagle, 2022). While participants uniformly condemned sexual relationships with prisoners, they held differing views on what it means to show compassion and where boundaries should be drawn.

The emotions that it evokes in them [uniformed staff] and in me are different. It kind of clashes. It's the emotional component that is really hard. I get very frustrated that the things I’m viewed as doing, whether I’m having a meeting with someone, spending more time with an inmate, running groups, or helping them out. I have been called a con lover by staff, which is very frustrating because that has a lot of connotations that I don’t like. That's just who I am as a person; caring, nurturing, wanting to support individuals the best I can. It has been emotionally very difficult. (Brittany, nurse)

Brittany highlights what Ashforth and Kreiner (1999) identify as the social taint that is borne from occupations that require regular contact with, or being servile to, people from stigmatized groups. For a guard to denigrate a nurse for showing compassion toward patients *because* they are criminalized creates a toxic work environment and demonstrates the lack of respect that nonuniformed staff experience from their security colleagues. Ievins et al. (2019, 2023) uncovered that prison officers subscribe to one of two penal philolophies, what she describes as the thin and thick custodial models; the first requires professional moral detachment and impartiality, while the second led officers to want the power to prolong a prisoner's detainment if they are not believed to have truly transformed. Officers who subscribed to the thick professionalism model were typically older and more experienced: “At their best, they managed to acknowledge prisoners’ offences without reducing them to them… at their worst, this knowledge led them to dangerously overuse their power” (Ievins, 2019: 104).

This power was not only expressed in relation to their interactions with prisoners but also with fellow staff members whose interactions with prisoners they actively surveilled (Ievins, 2019, 2023). The most frequently referenced fear that nonuniformed staff reported regarding uniformed staff viewing them to be “con lovers” was that they would think that they are engaging in an inappropriate relationship with a prisoner, the highly gendered connotation being that the inappropriateness is of a sexual nature.

There was an opportunity to have photos taken and a couple of guys in the group wanted pictures with us…In my picture with [], I made a mistake and realised it after the fact. I had my hands up a bit, so it looks like we were hugging… one of the staff members was clearly unimpressed. I should have been able to maintain professionality and take that step back and remember the environment we were in… I want to be mindful of the impression I’m giving. It could be this innocuous thing, like with the photo or writing a note to someone, that on the outside, it's not that big of a deal, but you put it in this environment [where] you have different people watching and the different impressions that can cause. Even if the things that we’re writing are completely appropriate, it doesn’t necessarily matter what the intent is, *it's about the optics of it*. (Elaine, volunteer)

Every participant reported “hearing stories” about cases where a staff member was accused or found guilty of engaging in inappropriate sexual relationships with a prisoner, despite being warned about such risks during training. Amy (programs officer) discussed the New Employee Orientation Program where staff are taught “how to be careful. And because [prisoners] can manipulate you, you get a lot of training about [how] you can be a sitting duck for manipulative inmates…so they teach you about boundaries and professionalism.” Amy further explained that every move she makes is “constantly monitored” by other staff.

I had a colleague that I did not like [who] tried to … put me under the bus and insinuate that I was being too nice to this offender. So, you’ve got staff ratting on each other. It's crazy…It's a quasi-military environment that way, you got to be careful… It's a real dance because you want to get along with the guys, but you can’t look like you’re really enjoying it.

Amy reveals the degree of surface acting (Hochschild, 1979, 1983) that staff engage to avoid being perceived as too friendly with prisoners. This can be a difficult boundary to determine and is specific to each staff–prisoner dynamic, what the staff member knows about the prisoner's institutional history and convictions, the staff member's interpretation of their responsibilities in terms of providing care (Tait, 2008, 2011), and their approach to professionalism (Ievins, 2019, 2023). Kate (parole and programs officer) similarly recounted:

I never allowed inmates in my office. Whenever I [did] interviews, I would do them in the classrooms in the units where they were locked up. For one, I liked my office, and I didn’t want inmates in my personal space. Secondly, it was isolated, and the guards didn’t come there very often… I thought of it as a safety issue for myself. I also thought it was *bad optics*. You know, a female staff meeting with inmates alone in her office way off in some corner of the institution. It just doesn’t look good. You always think of staff compromise. And I didn’t want to set myself up in any situation where an inmate thought he could try to trick me into something. I was very protective of that space and my safety that way and how things looked.

Kate's experience speaks to two concerns that are born from the risk logic that contours the occupational culture in carceral spaces—the first being the “bad optics” of a female staff member having a meeting with a prisoner in her office, the second being the fear that a prisoner may either harm or try to manipulate her in that space. Ievins (2019, 2023: 119) similarly found that “concerns about potential sexual manipulation” led COs to see interactions with prisoners convicted of sexual offences as sources of risk to be avoided and policed, with female officers being especially subject to “lateral surveillance.” Such concerns reveal the gendered nature of the stain that female staff who express compassion are hazarding simply by doing their jobs. It also reveals the degree to which staff have internalized self-discipline to uphold the feeling and display rules of the organization's emotion culture. As Jane (CO and parole officer) stated, “as a woman working in there, it's hard. Staff think you’re too nice… you gotta stand your ground, but not too strong, cause then you’re bitchy… Mentally, it's exhausting.” As Ashforth and Kreiner (1999: 415) contend, “the common denominator amongst tainted jobs is not so much their specific attributes but the visceral repugnance of people to them.”

One of the ways that compassion operates to heighten staff differences is that it exists in tension with the emotions that are attached to prisoners. Indigenous staff felt this tension deeply noting they must manage their behaviors and emotions more than others because Indigeneity is always-already saturated with feelings of suspicion and fear. As Jason, an Indigenous Liaison Officer stated, “being Indigenous, there's a lot of people within corrections that think that we can be compromised easily by the Indigenous offenders, like they lack trust in us to do our roles.” Jason stated that for Indigenous staff, such as Elders, outward displays of compassion like hugging *is* the norm.

In Indigenous corrections and interventions, it's a little bit different, especially with the Elders, because the Elders are very hands on with the offenders, meaning that they like to give them hugs and congratulate them when they get good news and express empathy when something ugly happens for these guys and the offender is crying.

Jason felt that he must police his behavior and carefully balance his work with being human.

Indigenous CSC staff…we don’t hug, we shake their hand… we extend our hand as a goodwill gesture… expressing gratitude and whatnot. I have hugged an offender. I can’t remember why, but it's really not encouraged that we do that as staff members, because a lot of people may start talking about it. Upper management may start talking about it. They may wonder if I’ve been compromised or my colleague has been compromised. I tend to have a little bit of a barrier bubble between [me and the prisoners].

For Jason, the tension between these two competing emotional requirements made it difficult to be himself as an Indigenous person. On one hand, Indigenous teachings require compassion to move people toward healing (Krawec, 2022), while on the other, CSC culture interprets these acts as evidence of being a compromised and untrustworthy employee. Jason's attempts at being a “true carer” (Tait, 2008, 2011) are seen as evidence of being compromised, which changes the way he cares for prisoners—leading him to be more distant and to follow the institutional expectation for professional detachment (Elliott, 2007; Ievins, 2019, 2023; Tait, 2008, 2011) rather than expressing the more open displays of compassion he initially sought to perform.

Security staff understand the emotion work of showing compassion via empathy and kindness to prisoners as a risk because it could signal that the staff member was hoodwinked by manipulative prisoners, which can lead to clashes between uniformed and nonuniformed staff. The assumption that prisoners are inherently manipulative is a common prejudice, especially among security staff (Crawley, 2004a, 2004b; Ievins, 2019, 2023), but this bias is antithetical to the professional approach that nonuniformed staff—especially health care workers—must maintain to successfully perform their work.

There's times where an inmate will say, “you got your haircut, it looks nice.” Now, on an operational side, that's instantly devious. They are grooming you. Whereas I respond by saying, “thank you. Yes, I got it done last week.” That is a prosocial interaction. That is not inappropriate. (Brittany, nurse)

Ievins (2023: 119) found that “anxious officers saw the signs of sexual grooming in even the most innocuous communications” such as wishing staff a merry Christmas or to have a good weekend, which suggests that anyone who is too closely linked with someone convicted of a sexual offence is “corrupted by association.” Such a discourse suggests that “the offenses ‘sex offenders’ have committed should shape the way everyone interacts with them.” They have lost their claims to full humanity, and with them, their right to be treated the same as other prisoners and other citizens (Ievins, 2023: 104). Brittany's narrative illustrates a central difference in how uniformed versus nonuniformed staff view the humanity and personal motivations of individual prisoners. Born from their training and responsibility to assess and mitigate all possible risks, rather than assuming the individual prisoner is offering a compliment as a normal part of human social interaction, uniformed staff tend to assume the worst. Not only does this suggest a dark prejudice among security staff but it also presupposes that nonuniformed staff are inherently naive about their professional working relationships with prisoners who they alternatively identify as “clients” or “patients.”

Nonuniformed staff consistently reported that they felt the need to “justify” why they had chosen to do work that supports incarcerated people to their uniformed colleagues, illustrating the repugnance described by Ashforth and Kreiner (1999). If we accept that perceptions of dirt as matter out of place are contextual (Douglas, 1966/2002), we can better understand how the risk logic of the carceral environment shapes these perceptions in ways that require continuous taint management by staff who do support work that requires expressions of compassion. As participants noted, this is a “delicate balancing act,” “a negotiation” that requires “juggling” or simultaneously “wearing different hats,” which is “mentally exhausting.”

## Conclusion

This article challenges compassion's reputation as an inherently “good” emotion. We explored how compassion operates in the emotional arena of the prison with a unique focus on the experiences of nonuniformed staff. Correctional Service of Canada's culture, norms, and values, what we termed the security-care nexus, encourages staff to perform a compulsory compassion that does not allow staff to be moved by prisoner suffering or to express *feeling* for incarcerated people, instead requiring a detached demeanor and strict institutional rule adherence that characterize Ievins (2019, 2023) thin custodial model. These compulsory compassionate performatives widen the social gap between those who give and receive compassion and reveals the “microhierarchical arrangements” (Clark, 1997) that impact staff dynamics and reinforce punitive power relations. This dichotomy was strongly felt by nonuniformed staff, who showed compassion to prisoners by way of hugs and engaging in personal conversations, only to be regarded as suspicious “con huggers/lovers” by their uniformed colleagues. Compassion works to bind the disgust and fear attached to prisoners to staff who resist institutional norms that emphasize punishment by showing compassion. Being identified as “con huggers/lovers” pushes nonuniformed staff to fall in line with the prevailing security ethos that structures the institutional norms of this setting, which was shown to lead to mental fatigue and stress for nonuniformed staff.

The daily pressure to perform their work in an emotionally detached way (Tait, 2008, 2011) is antithetical to the disciplinary training most nonuniformed staff receive and which emphasizes the importance of expressing care to develop rapport with patients and clients. Nurses and counselors in particular expressed concern about the difficulties they faced with respect to the judgement they feel by their security colleagues for expressing compassion to incarcerated people and how this requires taint management strategies and emotion work to save face. The emotion culture of this environment subsequently shapes and stifles how compassion is performed and thus the potential impact that it might have in the rehabilitative process. To promote the healing that is required for rehabilitation, health and other care work must be removed from the purview of correctional authorities and reintegrated into community settings, which would help overcome the microhierarchical arrangements that strain staff relationships.

## Notes

1. Virtual (e.g., Zoom or phone) interviews were conducted due to the COVID-19 pandemic and public health lockdown orders in place at the time.

2. Following CSC’s designation, ‘volunteers’ include participants who work as members of prisoner support groups, chaplaincy, recreation activities, classroom and workshop instruction and cultural activities. Volunteers are unpaid, but receive correctional training before working in that space and have daily interactions with correctional employees that acclimatizes them to the culture of this emotional arena.

3. Some participants held multiple staff roles throughout their employment with CSC.

4. Healing lodges are designed for Indigenous prisoners and offer cultural services and programs that incorporate Indigenous values, traditions and beliefs. Interventions include Elder services, spiritual leadership and cultural ceremonies. Critics problematize the pan-Indigenous approach and correctional interpretations of Indigenous risk (Martel et al., 2011).
